# Establishment of HK-2 Cells as a Relevant Model to Study Tenofovir-Induced Cytotoxicity

**DOI:** 10.3390/ijms18030531

**Published:** 2017-03-01

**Authors:** Rachel A. Murphy, Reagan M. Stafford, Brooke A. Petrasovits, Megann A. Boone, Monica A. Valentovic

**Affiliations:** 1 Department of Pharmacology, Physiology, and Toxicology, Joan C. Edwards School of Medicine, Marshall University, Huntington, WV 25755, USA; murphyrachel63@gmail.com (R.A.M.); stafford34@marshall.edu (R.M.S.); bpetrasovits2010@aol.com (B.A.P.); 2 West Virginia Wesleyan College, Buckhannon, WV 26201, USA; boone.195@osu.edu

**Keywords:** tenofovir, tenofovir disoproxil fumarate, nephrotoxicity, Fanconi Syndrome, HK-2 cells

## Abstract

Tenofovir (TFV) is an antiviral drug approved for treating Human Immunodeficiency Virus (HIV) and Hepatitis B. TFV is administered orally as the prodrug tenofovir disoproxil fumarate (TDF) which then is deesterified to the active drug TFV. TFV induces nephrotoxicity characterized by renal failure and Fanconi Syndrome. The mechanism of this toxicity remains unknown due to limited experimental models. This study investigated the cellular mechanism of cytotoxicity using a human renal proximal tubular epithelial cell line (HK-2). HK-2 cells were grown for 48 h followed by 24 to 72 h exposure to 0–28.8 μM TFV or vehicle, phosphate buffered saline (PBS). MTT (MTT, 3-(4,5-dimethylthiazol-2-yl)-2,5-Diphenyltetrazolium Bromide) and Trypan blue indicated that TFV diminished cell viability at 24–72 h. TFV decreased ATP levels at 72 h when compared to vehicle, reflecting mitochondrial dysfunction. TFV increased the oxidative stress biomarkers of protein carbonylation and 4-hydroxynonenol (4-HNE) adduct formation. Tumor necrosis factor alpha (TNFα) was released into the media following exposure to 14.5 and 28.8 μM TFV. Caspase 3 and 9 cleavage was induced by TFV compared to vehicle at 72 h. These studies show that HK-2 cells are a sensitive model for TFV cytotoxicity and suggest that mitochondrial stress and apoptosis occur in HK-2 cells treated with TFV.

## 1. Introduction

Tenofovir disoproxil fumarate (TDF) is a nucleotide reverse transcriptase inhibitor that is approved in the United States to treat Human Immunodeficiency Virus (HIV) and chronic Hepatitis B. Tenofovir (TFV) is administered as the prodrug TDF; TDF was designed to improve the bioavailability of TFV by the addition of 2-methyl carbonate esters which are deesterified to the active drug TFV [1]. TFV is efficacious to both treatment-naïve and treatment-experienced patients and has been beneficial in patients that have acquired resistance to other HIV medications [2,3]. TFV is considered a first line agent essential for treatment of HIV by the World Health Organization (WHO Model List 2015). TFV is the only antiretroviral that is a nucleotide analogue reverse transcriptase inhibitor. Compliance is higher with TFV than with other antiretroviral agents due to fewer side effects and once daily dosing [4].

Despite many positive therapeutic attributes, an adverse effect of TFV is nephrotoxicity. TFV-induced nephrotoxicity is characterized by a decreased glomerular filtration rate (GFR), increased serum creatinine, renal failure, and Fanconi-like Syndrome [5,6]. Fanconi Syndrome is a proximal tubular transport abnormality associated with impaired tubular reabsorption resulting in excess glucose, protein, urate, and phosphate excretion.

Renal excretion requires both glomerular filtration and tubular secretion. TFV is transported from blood into proximal tubular epithelial cells by organic anion transporters 1 and 3 (OAT1, OAT3, respectively) [7,8]. Renal toxicity was diminished in OAT1 knockout mice, indicating that OAT1 has a primary contribution to TFV renal uptake into proximal tubular epithelial cells [7]. In vitro studies reported that HEK293 cells transfected with OAT1 were much more sensitive to TFV cytotoxicity than cells transfected with OAT3 [9]; further, intracellular TFV concentrations were higher in HEK-OAT1 transfected cells compared to wild type and these findings support the concept that OAT1 is important for TFV uptake. The drug can then undergo tubular secretion into the lumen via the multidrug resistant proteins 2 (MRP2) and 4 (MRP4) [7,10]. While there is knowledge of the mechanisms contributing to tubular transport, the mechanism of TFV-associated proximal tubule damage is not extensively known at this time. This knowledge gap is due largely to limited experimental models; in vivo studies have used TFV treatment for 3 to 8 weeks [11,12], or primary renal cells exposed for up to 22 days [13]. The long duration of these experiments is not ideal and can hamper mechanistic studies. Additionally, daily treatment of rats for an extended period can cause significant stress, adding other complications to a mechanistic study. Cell lines can circumvent the contribution of stress as well as eliminate extrarenal factors and hemodynamics.

The purpose of this study was to explore the in vitro cytotoxicity of TFV in HK-2 cells. We selected Human Kidney 2 (HK-2) cells as a model as these cells are an adult, noncancerous, immortalized human epithelial cell line. HK-2 cells maintain biochemical properties and activity similar to in vivo proximal tubule cells [14,15,16]. Additionally, HK-2 cells express OAT1 and OAT3 proteins [17], which facilitate TFV transport into proximal tubule cells.

In this manuscript, we demonstrate that the HK-2 cell is a viable model to study TFV-induced cytotoxicity at clinically relevant concentrations. Further, these studies revealed that oxidative stress, induction of apoptosis, and mitochondrial decline in ATP are associated with TFV renal cytotoxicity.

## 2. Results

### 2.1. TFV Effects on Cell Viability

**Figure 1:**
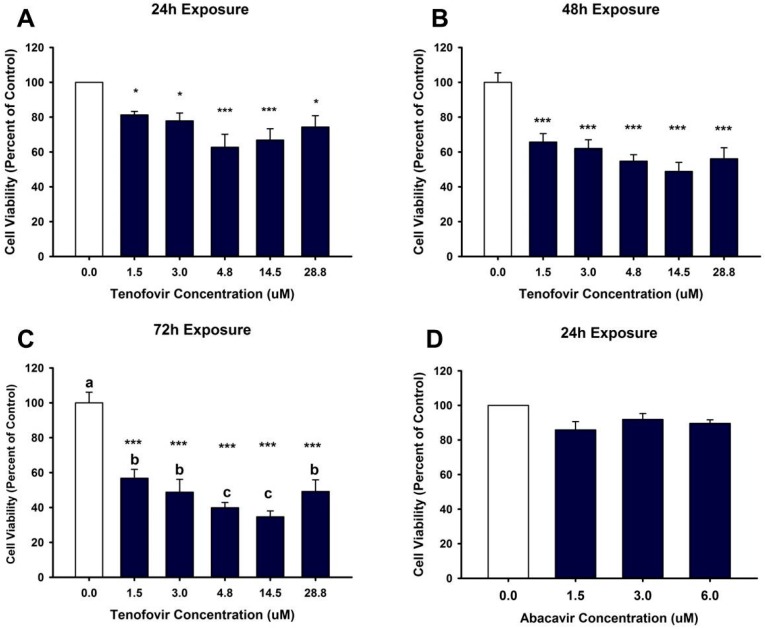
Tenofovir cytotoxic effects on cell viability in HK-2 cells using MTT (MTT, 3-(4,5-dimethylthiazol-2-yl)-2,5-Diphenyltetrazolium Bromide). Tenofovir diminished cell viability at 24 (**A**), 48 (**B**) and 72 h (**C**). Statistical difference from 0 μM tenofovir depicted in (**A**,**B**) by asterisks (* *p* < 0.05, *** *p* < 0.001). Different superscript letters (**C**) indicate groups that are different from each other (*p* < 0.05); (**D**) depicts cell viability following exposure for 24 h to another antiviral agent, abacavir. Abacavir did not alter viability at the concentrations tested. Each bar represents Mean ± SEM for three independent experiments run with two biological replicates.

**Figure 2:**
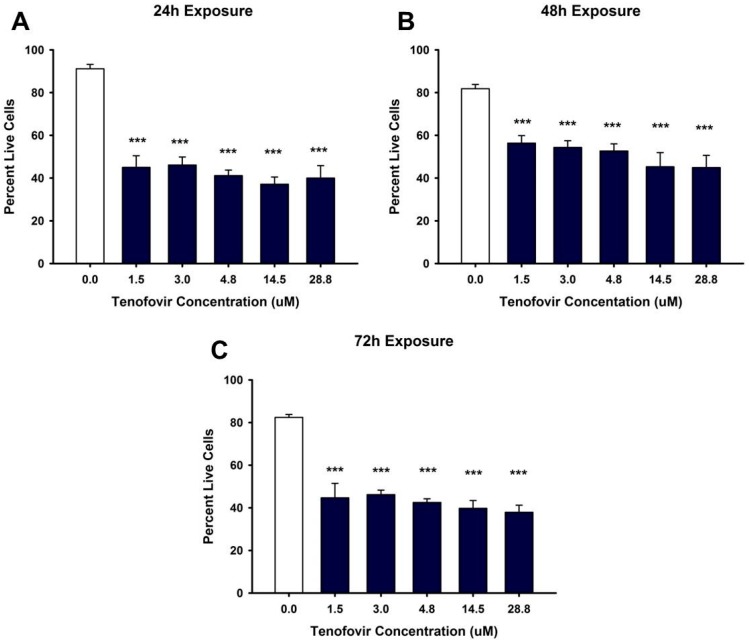
Tenofovir cytotoxic effects on cell viability in HK-2 cells using Trypan Blue Exclusion. Tenofovir diminished cell viability at 24 (**A**), 48 (**B**) and 72 h (**C**). Statistical difference from 0 μM tenofovir is denoted by asterisks (*** *p* < 0.001). Each bar represents Mean ± SEM for three independent experiments run with two biological replicates.

Based on the MTT (MTT, (3-(4,5-dimethylthiazol-2-yl)-2,5-Diphenyltetrazolium Bromide)) assay, TFV reduced cell viability within 24 h (*p* < 0.05) at all tested concentrations (Figure 1). MTT values continued to show diminished cell viability at 48 and 72 h (*p* < 0.001) for all TFV concentrations when compared to vehicle control (Figure 1). TFV final concentrations of 4.75 and 14.5 μM showed additional decline in cell viability at 72 h compared to other treatment groups (*p* < 0.05) (Figure 1). Trypan blue exclusion and cell counts were conducted in order to confirm that the TFV mediated decline in formazan formation could not be attributed to a direct effect of TFV on mitochondrial reductase enzymes converting MTT to formazan. Trypan blue cell counts (Figure 2) confirmed that TFV diminished cell viability beginning at 24 h and continuing for 72 h when compared to vehicle control. There was no difference in cell viability between groups. IC_50_ values calculated using MTT data were 9.21 and 2.77 μM TFV at 48 and 72 h, respectively. MTT viability studies conducted with the less nephrotoxic antiviral agent, abacavir, indicated that abacavir did not reduce cell viability at 24 h (Figure 1D) suggesting that our model is an appropriate model to examine nephrotoxic agents. Further experiments were initiated with TFV to explore the cellular mechanism of TFV cytotoxicity.

### 2.2. TFV Effects on Mitochondrial Function

**Figure 3:**
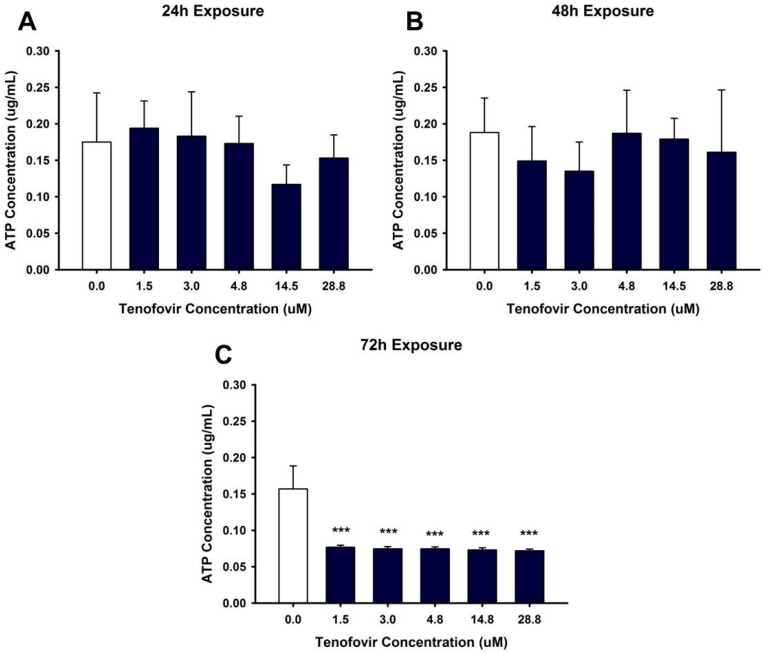
Tenofovir effects on ATP levels in HK-2 cells. ATP levels were measured following 24 (**A**), 48 (**B**) and 72 h (**C**) exposure to tenofovir. ATP was measured using an ATP Assay kit (Biovision) and simultaneously run ATP standards. Statistical difference from 0 μM tenofovir denoted by asterisks (*** *p* < 0.001). ATP levels at 24 and 48 h represent Mean ± SEM for two independent experiments run with three biological replicates. ATP levels at 72 h represent Mean ± SEM for four independent experiments run with three biological replicates.

Our hypothesis was that TFV impairs mitochondrial function resulting in decreased ATP levels. ATP levels were unchanged compared to vehicle by TFV after 24 or 48 h incubation (Figure 3). ATP levels were diminished by all concentrations of TFV at 72 h when compared to vehicle (*p* < 0.001) (Figure 3). These results suggest that multiple mechanisms of cytotoxicity may be induced by TFV since the reduction in ATP levels was not apparent prior to a reduction in cell viability.

### 2.3. TFV Effects on Oxidative Stress

**Figure 4:**
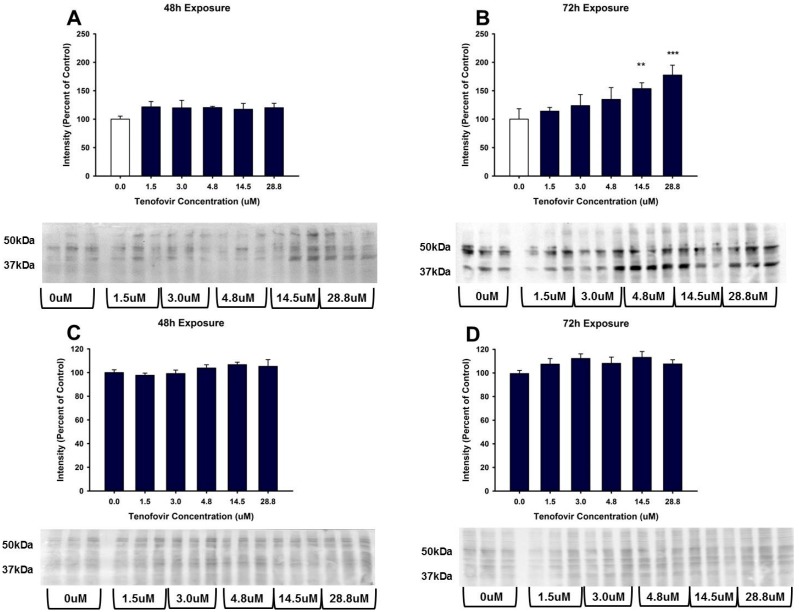
Tenofovir treatment for 72 h increased protein carbonylation in HK-2 cells. Protein carbonylation was measured in HK-2 cell lysate following 48 (**A**) and 72 h (**B**) exposure to TFV. Representative gel and cumulative densitometry included in each panel. Representative blot with MemCode Reversible staining for equal 25 μg protein loading and cumulative protein densitometry depicted for 48 (**C**) and 72 h (**D**) exposure. Asterisks (** *p* < 0.01, *** *p* < 0.001) indicate statistical difference from the vehicle control group. Each bar represents Mean ± SEM for three independent experiments run with three biological replicates.

**Figure 5:**
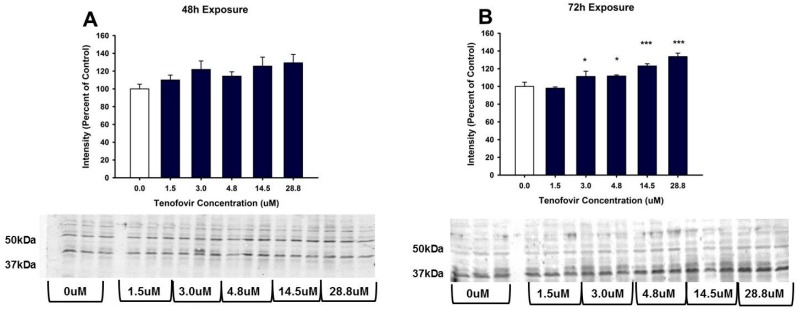

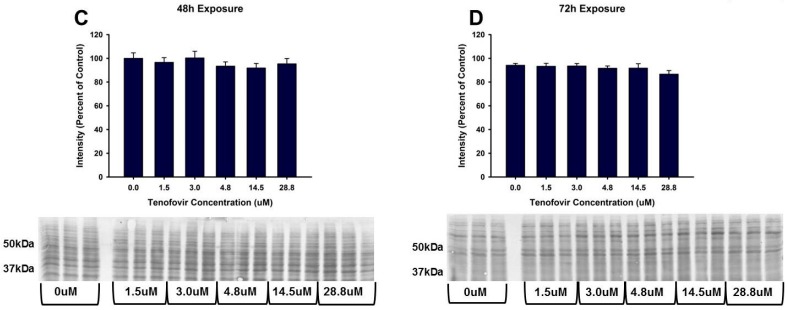
Tenofovir effects on 4-HNE adduct formation in HK-2 cells. 4-HNE adduct formation was measured in HK-2 cell lysate following 48 h (**A**) and 72 h (**B**) exposure to TFV. Representative blots and cumulative densitometry included in each panel. Representative blot with MemCode Reversible staining for equal 40 μg protein loading and cumulative protein densitometry depicted for 48 h (**C**) and 72 h (**D**) exposure. Asterisks (* *p* < 0.05, *** *p* < 0.001) indicate statistical difference from the vehicle control group. Each bar represents Mean ± SEM for three independent experiments run with three biological replicates.

**Figure 6:**
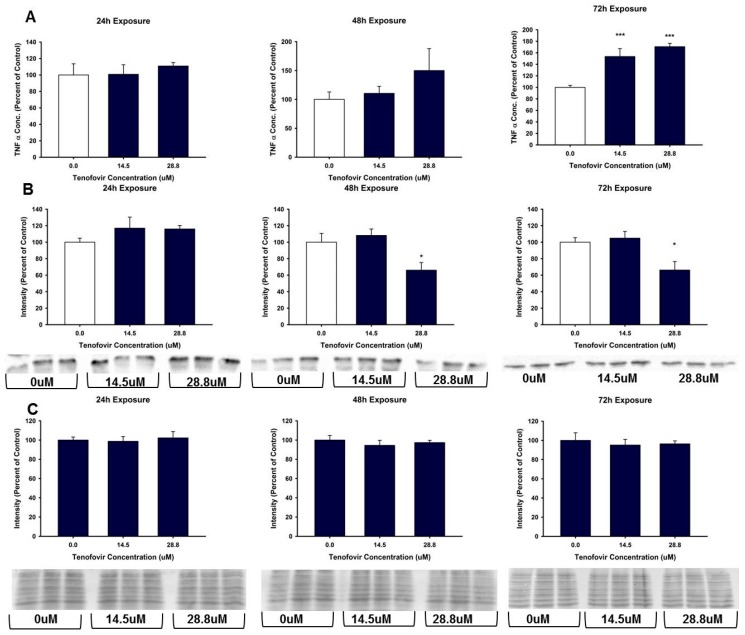
TNFα expression in HK-2 cell lysate and media following tenofovir exposure. (**A**) TNFα release into media expressed as a percent of control following 24, 48, and 72 h TFV exposure. TNFα in media was increased following a 72 h exposure for all concentrations relative to control (*** *p* < 0.001); (**B**) provides representative blot and cumulative densitometry for TNFα expression in cell lysate following 24, 48, 72 h TFV exposure. Statistical difference from 0 μM tenofovir depicted by asterisk (* *p* < 0.05); (**C**) depicts cumulative densitometry and representative blots for 50 μg protein loading of cell lysates visualized with MemCode Reversible Protein staining. Each bar represents Mean ± SEM for two independent experiments run with three biological replicates.

TFV increased oxidative stress as shown by Oxyblot analysis at 72 h relative to control in groups treated with 14.5 and 28.8 μM TFV (*p* < 0.001, Figure 4). Protein carbonylation was not increased relative to control at 48 h or at lower TFV concentrations at 72 h. 4-HNE adduct formation was increased following 72 h exposure for treatment groups of 3.0 to 28.8 μM TFV (Figure 5). TNFα secretion into the media was increased at 72 h exposure to TFV compared to vehicle (*p* < 0.001). TNFα expression in cell lysate was decreased at 48–72 h (*p* < 0.05) as shown by Western blot (Figure 6). These results suggest that TNFα is released into the media as TFV cytotoxicity occurs and that TFV does not increase TNFα protein expression. TNFα expression in the TFV treated cells was similar to vehicle control at 24 h when cell viability was diminished. These results suggest that it is unlikely that TNFα is the primary event in the mechanism of TFV cytotoxicity but rather occurs as a result of initial toxicity. The subcellular generation of oxidative stress may be due to mitochondrial damage, as ATP levels were also diminished by TFV. It is still not clear if TFV directly damages the mitochondria, leading to oxidative stress, or if TFV induces reactive oxygen species that subsequently damage mitochondria; additional studies are needed to further explore the mechanism of cytotoxicity. 

### 2.4. TFV Effects on Apoptosis Initiation

**Figure 7:**
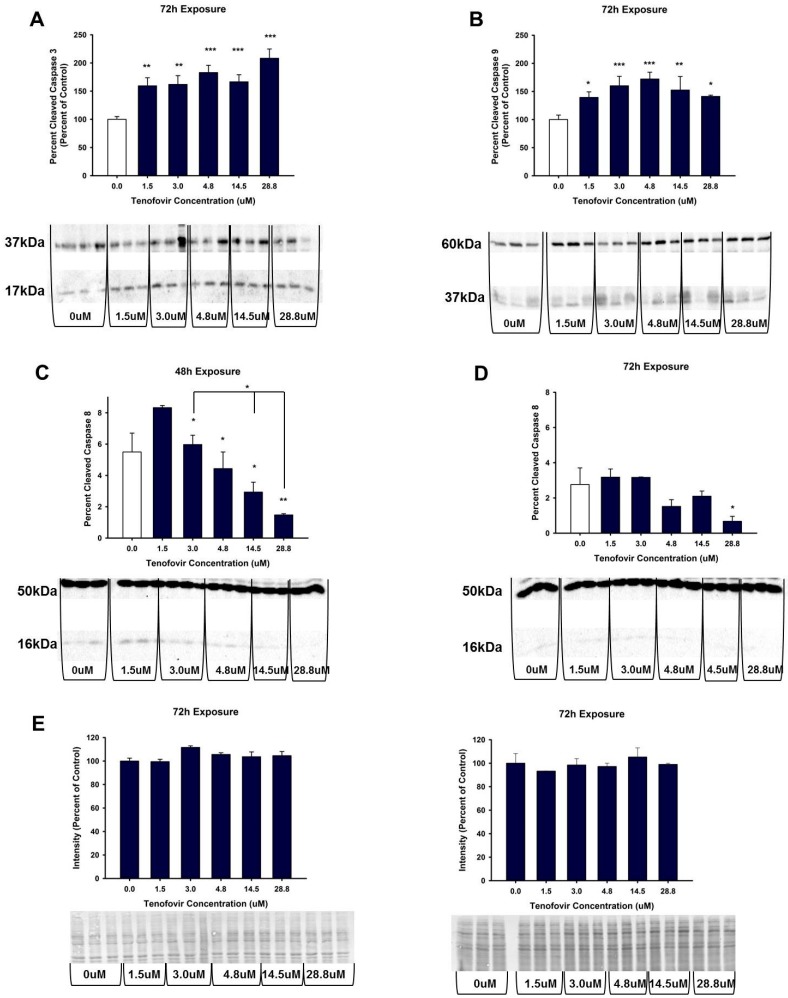

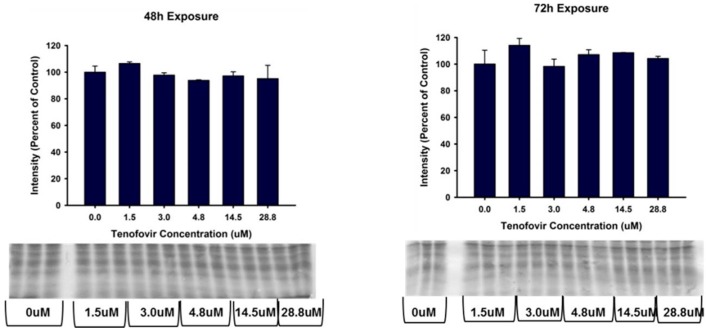
Tenofovir effects on the expression of cleaved caspase 3, 8 and 9 in HK-2 cells. Representative blot and cumulative densitometry for cleaved to total caspase 3 (**A**), caspase 9 (**B**) and caspase 8 (**D**) protein expression following 72 h. Asterisks (* *p* < 0.05, ** *p* < 0.01, *** *p* < 0.001) indicate statistical difference from the vehicle control group; (**C**) Depicts 48 h caspase 8 cleavage. (**E**) four representative blots of MemCode reversible staining for 40 μg protein loading and cumulative densitometry; blots and densitometry depicted in **E** correspond to: (top left is for **A**) and (top right **B**) while (bottom left is representative blot and densitometry for protein loading in **C**) and (bottom right is protein loading and representative blot for **D**). Each bar represents Mean ± SEM for three independent experiments run with three biological replicates.

TFV increased cleaved caspase 3 (*p* < 0.0.05) and cleaved caspase 9 relative to control for all treatments at 72 h exposure (Figure 7). Caspase 8 expression was measured after 48 and 72 h exposure to TVF. TFV did not increase caspase 8 cleavage relative to control at either time point. Cleaved caspase 8 was minimal in all groups and was decreased by higher TFV treatment relative to vehicle control. These results suggest that the increase in TNFα in the media at 72 h was not sufficient to stimulate caspase 8 cleavage. These data show that TFV induces apoptosis in HK-2 cells, and that apoptosis is induced via mitochondrial damage.

### 2.5. Ascorbic Acid Protection of TFV Cytotoxicity 

**Figure 8:**
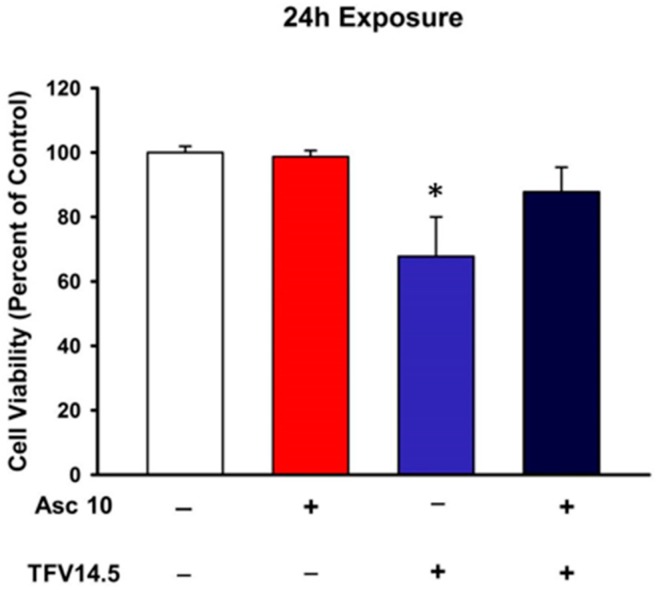
Effects of ascorbic acid on Tenofovir cytotoxicity. Ascorbic acid did not alter cell viability relative to vehicle control. Ascorbic acid protected HK-2 cells from tenofovir cytotoxicity as assessed by MTT viability. Cells were treated with 0 (−) or 10 µM (+) ascorbic acid. Followed by a 24 h exposure to 0 (−) or 14.5 µM TFV (+). An asterisk (* *p* < 0.05) indicates statistical difference from all other groups. Values represent Mean ± SEM for three independent experiments run with three biological replicates.

Oxidative stress was increased by TFV resulting in increased protein carbonylation (Figure 4) and increased 4-HNE protein adduction (Figure 5) suggesting that a rise in reactive oxygen species occurs with TFV cytotoxicity. Pretreatment with ascorbic acid reduced TFV toxicity in HK-2 cells (Figure 8) as cell viability was higher in cells exposed to TFV in the presence of ascorbic acid compared to TFV alone. These findings suggest that an antioxidant can reduce TFV cytotoxicity in HK-2 cells.

## 3. Discussion

TFV is a very effective antiviral nucleotide reverse transcriptase inhibitor prescribed worldwide in the treatment of HIV and Hepatitis B. Patients treated with TFV have an increased incidence of renal impairment [18,19]. Renal TFV toxicity in humans is characterized by Fanconi Syndrome, and depending on the dose, development of irreversible impaired renal function. There is a positive correlation between increased TFV plasma concentration, renal toxicity and the duration that patients were treated with TFV [20,21]. Because treatment with TFV is chronic, examination of the mechanism of renal toxicity is clinically relevant. Understanding the mechanism is essential for the development of methods to mitigate TFV renal impairment.

Mechanistic studies require a model that is consistent with what occurs in humans. The selection of suitable models to study TFV cytotoxicity has been problematic as most in vivo models require subchronic treatment of animals. TFV toxicity in rodents such as rats has required 8 weeks of daily treatment in order to develop nephrotoxicity [11]. In this study, rats treated for 8 weeks exhibited diminished renal function and proximal tubular damage along with enlarged mitochondria. Another study showed that treatment for 5 weeks with TFV induced oxidative stress in Wistar rats dosed with very high levels of TFV [22]. 

An in vitro model was first reported by Wang and Flint (2013) that developed TFV cytotoxicity using primary human kidney cells [23]. Primary human kidney cells required culturing for 19 days with 200 μM TFV to induce cytotoxicity, which was a long duration at concentrations higher than pharmacological levels. The need for higher concentrations of TFV to induce toxicity in primary proximal tubule cell lines may be due to differences in OAT1 and OAT3 expression. Endogenous expression of OAT1 and OAT3 in primary human proximal tubule cells has been shown to vary greatly from sample to sample [24] and TFV-induced cytotoxicity is dependent on OAT1 and OAT3 expression [7,25]. Our HK-2 cell model is more sensitive than human primary proximal tubule cells as we have shown induction of cytotoxicity in 24 h compared to 22 days and at a concentration almost 10 times lower than reported for human primary proximal tubular epithelial cells [13]. In addition to inducing cytotoxicity at 24 h, our HK-2 cell model has an IC_50_ of 9.2 μM TFV at 48 h, and an IC_50_ of 2.77 μM TFV at 72 h. Other cell types such as HEK293 cells have been used to study tenofovir cytotoxicity, but appear less sensitive than our HK-2 model. One study showed that TFV-induced cytotoxicity within 48 h in HEK293 cells transfected with OAT1; however, the IC_50_ was reported to be 316 μM [9]. 

Our study is the first to show that HK-2 cells can be used to evaluate TFV cytotoxicity. TFV renal cytotoxicity was evident within 24 h at clinically relevant concentrations. Primary human kidney cells required a concentration of 200 μM, which is higher than reported maximal clinical steady state plasma concentration [23]. In comparison, in our study, viability was diminished in HK-2 cells with TFV concentrations ranging from 1.5 to 28.8 μM. The concentrations used in our current study are clinically relevant as plasma TFV concentrations have been reported to be 2.2 μM in HIV-1 infected patients [4,8]. Additionally, TFV plasma concentrations are higher in HIV-1 patients and non-infected individuals with existing renal impairment; patients with renal impairment also experience a longer duration of TFV exposure, as shown by a 15-fold increase in AUC_0–24h_ [21,26,27]. Therefore, HK-2 cells provide a model that can be used to examine the mechanism of toxicity without the compounding physiological parameters influencing the response of the kidney to a toxicant. Our studies further showed that HK-2 cells can differentiate the toxicity of different antiviral agents as abacavir, an agent that is considered less toxic to the kidney which was not toxic in our system (Figure 1D). 

It is probable that TFV renal cytotoxicity involves multiple mechanisms and that these mechanisms are not yet fully understood. TFV treatment of HK-2 cells decreased ATP levels when compared to control at 72 h exposure (Figure 3). Diminished ATP levels can be mediated by numerous pathways, including alterations in mitochondrial DNA stability, impaired mitochondrial function, or increased oxidative stress. 

Mitochondrial function relies on many factors, including the stability and integrity of mitochondrial DNA (mtDNA). Decreased mtDNA could result in a decline in cellular ATP levels. There are conflicting reports regarding the effects of TFX on mtDNA. The differences may be due to species variation and differences in experimental models. For example, TFV diminished mtDNA in HIV-1 transgenic mice [7] while a 19 day exposure of human primary renal proximal tubule cells to 2 and 200 μM TFV showed no change in mtDNA compared to control [13,23]. However, effects of TFV on mtDNA may vary between renal and nonrenal cells [23]. Additionally, mtDNA was diminished in human primary proximal tubule cells by another nucleoside reverse transcriptase inhibitor, adefovir, within 9 days of incubation with TDF [23], indicating that TFV may be a weaker inhibitor of DNA polymerase gamma. 

TFV increased expression at 72 h of cleaved caspase 3 and 9 in HK-2 cells (Figure 7). Apoptosis can be induced via many pathways, including extracellular signals and mitochondrial dysfunction. Each pathway activates different initiator caspase proteins; for example, cleavage of caspase 9 occurs when mitochondrial damage initiates the intrinsic apoptotic pathway. Initiator caspases then interact with other cellular proteins, forming an apoptosome that cleaves executioner caspases such as caspase 3 and activates the induction of cell death. The increased expression of cleaved caspase 3 and 9 in HK-2 cells treated with TFV indicated that apoptosis was occurring, and that mitochondrial dysfunction may be a major contributing factor to loss of cell viability (Figure 7). 

The renal proximal tubular epithelial cell has a high requirement for ATP generation to maintain normal cellular processes such as active transport. The decline in cellular ATP levels caused by TFV would impair normal proximal tubular epithelial cell function. TFV increased oxidative stress, as protein carbonylation was elevated at 72 h exposure to 14.5 and 28.8 μM TFV Figure 4. The HK-2 cells were more sensitive to TFV and displayed increased oxidative stress at clinically relevant concentrations as compared to previously examined models. A 5 week treatment of rats with 12 times higher dose than what is clinically used reported a 25% increase in renal protein carbonylation [22]; the HK-2 cells demonstrated a much greater increase in protein carbonylation within a shorter time period than reported in vivo treatment of rats (Figure 4), suggesting that HK-2 cells may be a more sensitive model to examine TFV cytotoxicity. 

TNFα is a proinflammaotry cytokine which is expressed in proximal tubular epithelial cells. TNFα can be secreted from cells to induce an inflammatory response as a protective mechanism during initial exposure to toxicants [28]. TNFα expression can induce reactive oxygen species generation via mitochondria and through activation of NADPH oxidase [29]. Mitochondrial damage and generation of reactive oxygen species can stimulate the release of various cytokines including TNFα [30]. These changes in TNFα expression and secretion can modulate various parts of the cellular antioxidant system and directly increase the production of mitochondrial reactive oxygen species through damage to the electron transport chain [30]. In experiments involving exposing HK-2 cells to toxicants and/or ischemic injury, TNFα levels rose along with many other markers of toxicity, including increases in oxidative stress via dysfunction of cellular antioxidant systems, activation of an inflammatory response and associated proteins, and induction of programmed cell death [31,32,33]. We examined TNFα as a potential indicator of oxidative stress. Our study showed that TNFα was released into the media at 72 h, while tissue showed a decline in expression at 48 and 72 h (Figure 6). Because TNFα expression was unaltered at 24 h (Figure 6) it is unlikely that activation of inflammatory response is the cause of the oxidative stress observed. The decline in TNFα in the cells is consistent with loss from cells into the media; TNFα released into the media may contribute to greater oxidative stress. Further studies need to explore the cellular mechanism for increased oxidative stress, TNFα secretion in HK-2 cells, specifically, evaluation of mitochondrial alterations that may mediate either a decline in mitochondrial function or increased reactive oxygen species.

## 4. Materials and Methods

### 4.1. Chemicals and Reagents

TFV was purchased from Cayman Chemicals (Item No. 13874, Ann Arbor, MI, USA) and was used for all studies. The vehicle for cell treatments was phosphate buffered saline (Invitrogen, Carlsbad, CA, USA, Item No. 14175095). MTT and other chemicals were purchased from Sigma Aldrich (St. Louis, MO, USA) or Fisher Scientific (Pittsburg, PA, USA). Antibodies and kits were purchased as indicated in the sections below. 

### 4.2. Cell Lines and Tenofovir (TFV) Treatment

Human immortalized epithelial HK-2 cells were purchased from the American Type Culture Collection (ATCC) and were cultured according to ATCC guidelines. Briefly, cells were grown in a keratinocyte-free media with 50 μg/mL bovine pituitary extract and 5 ng/mL recombinant epithelial growth factor from Invitrogen (Carlsbad, CA, USA, Item No. 17005-042). Cells were grown in a warm humidified incubator with constant settings of 37 °C and 5% CO_2_. HK-2 cells were plated into six-well tissue culture plates (750,000 cells/mL) (Corning, Sigma Aldrich Item No. CLS3516) and allowed to grow for 48 h. Media was replaced and cells were treated with a final concentration of 0, 1.5, 3.0, 4.75, 14.5, or 28.8 μM TFV for 24, 48, or 72 h. The vehicle was an equal volume of phosphate buffered saline (PBS). Abacavir was prepared in sterile water and cells were treated for 24 h with 0, 1.5, 3 or 6 μM of abacavir to evaluate renal sensitivity to an agent recognized to be less nephrotoxic. Following the treatment period, cells were collected with Trypsin-EDTA (0.25%) (Invitrogen, Item No. 25200072) for sample analysis. 

### 4.3. Cell Viability 

Cells were plated into 48-well tissue culture plates (39,000 cells/mL) (Cyto One, USA Scientific, Ocala, FL, USA, Item No. CC7682-7548) and allowed to grow for 48 h followed by treatment with vehicle or TFV (see Section 2.1). Following the treatment period, cell viability was assessed using the MTT assay [34]. The MTT assay relies on the conversion of tetrazolium dye 3-(4,5-dimethlthiazol-2-yl)-2,5-diphenyltetrazolium bromide (MTT) (Sigma Aldrich, Item No. M5655-5X1G) to formazan by NAD(P)H-dependent oxidoreductases. 

To ensure that results of the MTT Assay were not due to mitochondrial damage rather than cell death, a trypan blue exclusion test was run based on a previously published protocol [35]. In brief, an aliquot of collected cells are diluted 1:10 with 40% *w*/*v* Trypan Blue solution (Sigma Aldrich, Item No. T6146). The suspension was mixed via pipetting and allowed to sit for approximately 2 min. A 10 μL aliquot of suspension was transferred to a hemocytometer and total cells, live cells, and dead cells were manually counted. 

### 4.4. ATP Measurement 

ATP levels were assessed using the ADP/ATP Ratio Bioluminescence Assay Kit (Biovision Inc., Milpitas, CA, USA, Item No. K255-200). Briefly, 100 μL of reaction mix was plated into a 96-well plate (Corning, Sigma Aldrich, Item No. 356519) and allowed to establish a baseline during a 2 h room temperature incubation. A 10 μL aliquot of the sample was added to each well and bioluminescence was measured using a luminometer. Immediately following the measurement, 10 μL of diluted ADP-converting enzyme was added and bioluminescence was measured again. Lysate concentration of ATP and ADP was determined using a standard curve. 

### 4.5. Western Blot 

Western blot analysis was conducted to assess the expression of active caspase 3, 8 and 9 and the formation of 4-hydroxy-2-nonenal (4-HNE) protein adducts. Protein concentration in each sample was determined using the Bradford protein assay [36]. A 40 μg aliquot of each sample was denatured by boiling for 5 min. Proteins were then separated on a 12.5% polyacrylamide gel and transferred to a nitrocellulose membrane (Bio-Rad, Hercules, CA, USA, Item No. 1620115); successful transfer and protein loading were verified using MemCode Reversible Protein Stain Kit (Pierce Biotechnology, Rockford, IL, USA, Fisher Scientific, Item No. PI-24580). Membranes were blocked using either a 5% *w*/*v* milk/TBST solution (10 mM Tris-HCl, 150 mM NaCl, 0.1% Tween-20; pH 8.0) or a 1% Bovine Serum Albumin (BSA)/TBST solution for 1 h. Membranes were next incubated with continual shaking overnight at 4 °C with a rabbit polyclonal antibody for caspase 3 (1:1000 dilution, Cell Signaling Technology, Danvers, MA, USA, Item No. 9662), caspase 8 (1:400 dilution, Biovision Inc., Item No. 3020-100), caspase 9 (1:1000 dilution, Cell Signaling Technology, Item No. 9502) or 4-HNE (1:1000 dilution, Cell Bio Labs Inc., San Diego, CA, USA, Item No. STA-035) diluted in 5% milk/TBST or 1% BSA/TBST. The membranes were washed four times with TBST and goat anti-rabbit HRP-linked secondary antibodies were diluted to 1:5000 in 5% milk/TBST and added for 1 h. Membranes were washed again with TBST and then developed using Amersham ECL Western Blotting Detection Agent (GE Healthcare Life Sciences, Marlborough, MA, USA, Item No. RPN2232). A BioRad chemic-doc system was used to capture the gel image and used for densitometry analysis. 

### 4.6. Oxidative Stress

In addition to 4-HNE adduction of protein, another indicator of oxidative stress is protein carbonylation, which can produce an aldehyde or ketone side chain on amino acids. Protein carbonylation was analyzed using the Oxyblot Protein Oxidation Detection Kit (EMD Millipore, Billerica, MA, USA, Item No. S7150). Following treatment for 24–72 h with vehicle or TFV, cells were pelleted, rinsed with 500 μL PBS, and centrifuged. The pellet was then disrupted with lysis buffer. Protein content was measured, and a 25 μg aliquot was derivatized as previously described [37]. Protein carbonyl moieties on amino acids generated by oxidative stress are derivatized in the presence of 2,4-dinitrophenylhydrazine to stable 2,4-dinitrophenylhydrazone groups. The primary antibody recognizes 2,4-dinitrophenylhydrazone groups on proteins and was used at a dilution of 1:150. Protein loading was verified using the MemCode Protein Stain (see Section 2.5) and results were analyzed with BioRad Chemidoc densitometry software (version 4.0.1, Catalog No. 170-9690, BioRad, Hercules, CA, USA). A series of studies examined whether ascorbic acid, an antioxidant, could provide protection from TFV. HK-2 cells were allowed to grow for 48 h followed by a 1 h pretreatment with 0 or 10 μM ascorbic acid in sterile water followed by a 24 h incubation with 0 or 14.5 μM TFV. Viability of cells was assessed using the MTT assay. All media and MTT was removed from the cells prior to the addition of DMSO to prevent any interaction of ascorbic acid with formazan.

### 4.7. TNFα in Cell Media and Cell Lysate

Proximal tubular epithelial cells express TNFα. TNFα expression was measured in cell culture media using an ELISA assay kit and in cell lysate by Western blot. Release of TNFα into the cell culture media was measured using an ELISA kit (Abcam, Cambridge, MA, USA, Item No. ab181421) per the manufacturer’s instructions. Briefly, 50 μL of collected media and a capture/detector antibody cocktail were added to precoated wells and incubated for 1 h shaking at 400 rpm. Following the immunocapture incubation period, the wells were washed and TMB (TMB, 3,3′,5,5′-Tetramethylbenzidine) substrate was added, producing a color change based on the amount of bound TNFα, which was then read at 450 nm. TNFα concentration was determined using a standard curve. TNFα expression in TFV treated cell lysate was determined using Western blot as described above (see Methods 2.5). Each lane was loaded with 50 μg of protein; membranes were probed using and rabbit-polyclonal HRP-linked antibody for TNFα diluted to 1:1000 in 5% BSA/TBST (Abcam, Item No. ab66579). TNFα was normalized to protein and compared relative to control.

### 4.8. Statistical Analysis

Values represent Mean ± SEM with 2–4 independent experiments conducted with 2–4 biological replicates. Differences between groups were determined with a one-way ANOVA followed by a Holm–Sidak post-hoc test with *p* < 0.05 (SigmaStat, SSPS, Systat Software, Chicago, IL, USA, Item SigmaPlot SPW11).

## 5. Conclusions

HK-2 cells are a sensitive mode for examining TFV renal cytotoxicity. The concentrations of TFV used in these studies are clinically relevant. Intracellular concentrations of TFV can be higher than plasma levels as proximal tubule cells utilize active transport. TFV reduced cell viability within 24 h. This is the first report to characterize TFV toxicity using HK-2 cells. Our results show that TFV causes mitochondrial damage resulting in diminished cellular ATP levels and cleavage of caspase 3 and 9. Further studies are needed to explore the specific cellular mechanism of mitochondrial toxicity and oxidative stress. The study also showed that ascorbic acid, an antioxidant, could reduce TFV cytotoxicity.

## Abbreviations

TNFα	Tumor necrosis factor alpha
4-HNE	4-hydroxy-2-nonenal
HIV	Human immunodeficiency virus
MTT	3-(4,5-diemthylthiazol-2-yl)-2,5-dephenyltetrazolium bromide
MRP-2	Multidrug resistant protein 2
MRP-4	Multidrug resistant protein 4
OAT1	Organic anion transporter 1
OAT3	Organic anion transporter 3
TFV	Tenofovir
TDF	Tenofovir disoproxil fumarate
HK-2	Human kidney 2
GFR	Glomerular filtration rate
